# Multi‐scale mechanisms driving root regeneration: From regeneration competence to tissue repatterning

**DOI:** 10.1111/tpj.16860

**Published:** 2024-06-02

**Authors:** Monica L. García‐Gómez, Kirsten ten Tusscher

**Affiliations:** ^1^ Computational Developmental Biology Group, Department of Biology Utrecht University Padualaan 8 3584 CH Utrecht The Netherlands; ^2^ Experimental and Computational Plant Development Group, Department of Biology Utrecht University Padualaan 8 3584 CH Utrecht The Netherlands; ^3^ CropXR Institute Utrecht The Netherlands; ^4^ Translational Plant Biology Group, Department of Biology Utrecht University Padualaan 8 3584 CH Utrecht The Netherlands

**Keywords:** regeneration, root development, wound signaling, cell fate transitions, cell reprogramming, *Arabidopsis thaliana*

## Abstract

Plants possess an outstanding capacity to regenerate enabling them to repair damages caused by suboptimal environmental conditions, biotic attacks, or mechanical damages impacting the survival of these sessile organisms. Although the extent of regeneration varies greatly between localized cell damage and whole organ recovery, the process of regeneration can be subdivided into a similar sequence of interlinked regulatory processes. That is, competence to regenerate, cell fate reprogramming, and the repatterning of the tissue. Here, using root tip regeneration as a paradigm system to study plant regeneration, we provide a synthesis of the molecular responses that underlie both regeneration competence and the repatterning of the root stump. Regarding regeneration competence, we discuss the role of wound signaling, hormone responses and synthesis, and rapid changes in gene expression observed in the cells close to the cut. Then, we consider how this rapid response is followed by the tissue repatterning phase, where cells experience cell fate changes in a spatial and temporal order to recreate the lost stem cell niche and columella. Lastly, we argue that a multi‐scale modeling approach is fundamental to uncovering the mechanisms underlying root regeneration, as it allows to integrate knowledge of cell‐level gene expression, cell‐to‐cell transport of hormones and transcription factors, and tissue‐level growth dynamics to reveal how the bi‐directional feedbacks between these processes enable self‐organized repatterning of the root apex.

## Significance Statement

This review proposes a conceptual model of the molecular mechanisms underlying root tip regeneration, from regeneration competence to the spatio‐temporal repatterning of the root stump. We also discuss the use of multi‐scale modeling to address open questions in the field.

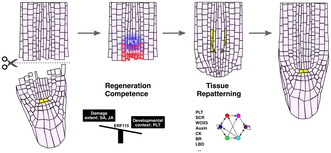

## INTRODUCTION

Plants possess an extraordinary capacity for regeneration that spans from repairing local cell damage to whole‐organ regeneration from tissue explants. Exposing plants to artificial damage in the laboratory has been a long‐established strategy to uncover the developmental mechanisms that allow them to respond, repair, and recover from damage. Frederick A. Clowes, the discoverer of the quiescent center (QC) in 1956, used maize (*Zea mays*) roots to show that these multi‐potent stem cells are more resistant to X‐ray‐induced DNA damage compared to other surrounding cells and that they are critical for root growth recovery after a 2‐week cold treatment (reviewed in Dubrovsky & Ivanov, 2021). Similarly, roots of Arabidopsis (*Arabidopsis thaliana*) seedlings exposed to freezing temperature (~4°) were shown to sacrifice their columella initial cells thereby increasing QC resilience to cold, while later restoring these lost cells from the surviving QC (Hong et al., 2017). Nowadays, the model species Arabidopsis is at the center stage of regeneration research allowing the discovery of important genetic and hormonal regulators of this process and the description of where and when are they active throughout the self‐organization of a new root apex.

In Arabidopsis research, stem cell and meristematic damages are mimicked by laser ablation of specific, localized cells (Hoermayer et al., 2020; Marhava et al., 2019; Smet & Blilou, 2023; van den Berg et al., 1997), as well as by using drugs like bleomycin that cause programmed cell death (Canher et al., 2020), or hydroxyurea that causes DNA stress (Cruz‐Ramírez et al., 2013). Such experiments have revealed that the regeneration capabilities of the root are not exclusively dependent on the QC. Instead, the repair of local damages relies on neighboring cells that in response to the damages experienced undergo cell fate reprogramming. Indeed, by ablating the QC without damaging the surrounding tissue, Arabidopsis roots were shown to be capable of generating a novel QC from the intact neighboring stele initials (Xu et al., 2006). Additionally, ablation of an individual cortex meristematic cell is rapidly repaired by divisions of the adjacent endodermal cell (Hoermayer et al., 2020; Marhava et al., 2019). Moreover, when instead bleomycin was applied to induce meristematic cell damage, again roots were found to be capable of specifically restoring the damaged tissue from the intact neighboring cells (Canher et al., 2020). Thus, while the QC cells are considered a reserve of multi‐potent stem cells capable of generating all root cell types and thereby able to repair damages in the meristem through damage‐induced reprogramming (Heyman et al., 2014), this regenerative potential is in fact broadly present in the meristem.

As the most spectacular example of root tip regeneration capabilities, in experiments where the entire root apex is resected, and with that the SCN and a considerable fraction of the meristem, a new root apex is regenerated in a matter of days. The higher the cut is performed, the less meristematic cells remain in the stump. This results in a lower regeneration success rate, revealing the existence of regulatory links between root developmental zonation and regeneration competence (Durgaprasad et al., 2019). If the root stump is regeneration competent, it will get repatterned through dynamic changes in cell fate of the cells remaining in the stump to form a new root apex (Efroni et al., 2016; Sena et al., 2009).

Regeneration in plants can even occur from tissue explants cultured *in vitro* in high auxin conditions to form a callus (Melnyk, 2023), which then, depending on the applied cytokinin‐to‐auxin ratio, will form shoots, roots, or so‐called somatic embryos (Christiaens et al., 2021; Sugimoto et al., 2019). Intriguingly, callus induction involves a root meristem‐like phase independent of both the future fate and the tissue origin of the explant (Kim et al., 2018; Sugimoto et al., 2010), necessary to grant regeneration competence (Kareem et al., 2015). Regeneration in the root is also characterized by the transient activation of root stem cell and embryonic programs, either to regenerate individual cells in the meristem (Marhava et al., 2019) or the whole apex (Efroni et al., 2016). While it remains to be explained why displaying root‐like characteristics enables the initial stages of these disparate examples of regeneration, this poses root regeneration as a paradigm system to uncover generic principles in plant regeneration.

Experimental research has uncovered the involvement of major root patterning regulators, wound‐induced signals, and epigenetic regulators in root regeneration. Despite this large and growing catalog of known players, we still have an incomplete understanding of how their combined activity is coordinated and integrated to enable root regeneration. More specifically, the mechanisms through which these factors together grant regeneration competence, guide cell fate reprogramming in the correct spatial order, and reconstitute the auxin transport network are incompletely understood. Based on the experimental details on root regeneration collected in the last two decades, here we propose a preliminary conceptual model of how key molecular regulators control consecutive and interrelated regulatory processes, where one sets the ground for the next to unfold. Finally, we argue that to put the regeneration puzzle together, multi‐scale computational models that enable the incorporation of the various involved processes, their different spatio‐temporal scales, and their feedback will be crucial.

**BULLET POINT SUMMARY**

1. A root meristem‐like phase characterizes the beginning of different instances of plant regeneration, posing root tip regeneration as a paradigm study system for regeneration.
2. The regeneration of the root apex involves two consecutive and interrelated processes: gaining competence to regenerate and tissue repatterning.
3. Root regeneration competence integrates the stemness and energy status of the cells remaining in the stump with molecular responses related to the extent of injury and potential pathogenic threats.
4. Tissue repatterning entails the activity of genetic–hormonal regulatory networks coupled with tissue‐level information to recreate the pattern of the root SCN.
5. Multi‐scale modeling is essential to integrate the different processes and scales involved in regeneration to fully uncover the mechanisms underlying root regeneration.

## SETTING THE STAGE: PATTERNING IN INTACT ROOTS

**Figure 1:**
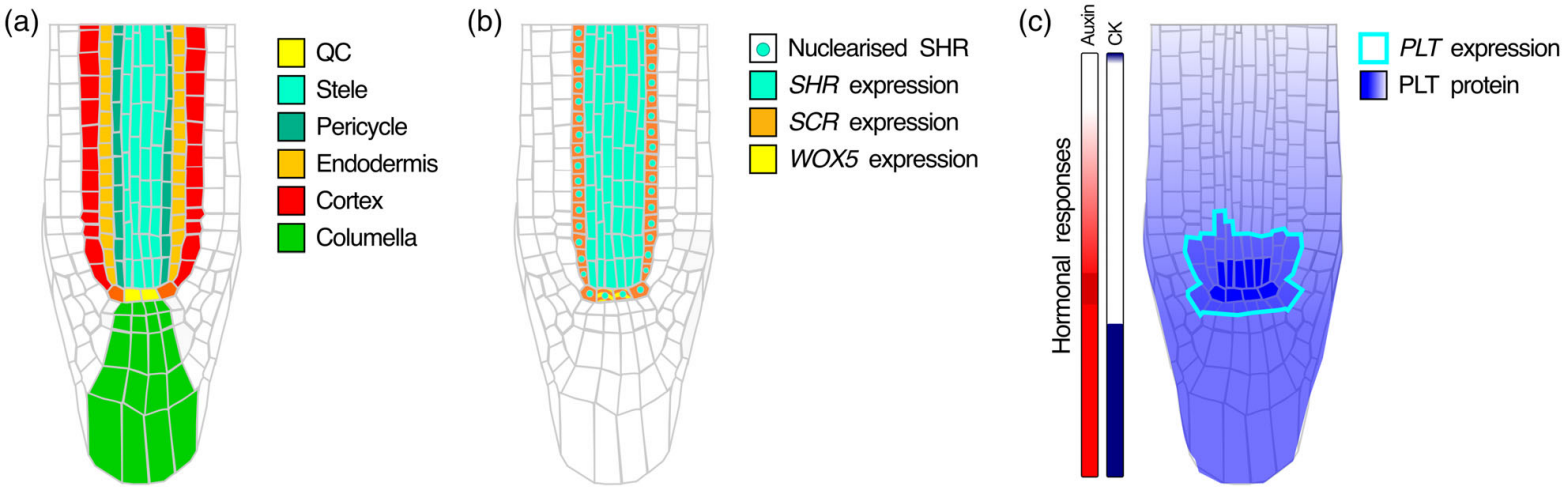
Cell types, gene expression, and hormonal distribution in intact roots. Cell types composing the root apex (a), molecular regulators of the radial (b), and longitudinal (c) patterning of the root meristem. CK, cytokinin; PLT, PLETHORA; QC, quiescent center; SCR, SCARECROW; SHR, SHORTROOT; WOX5, WUSCHEL‐related homeobox 5.

To introduce the major players, their spatial domain of activity, and the targeted end state of regeneration, here we first discuss the major aspects of root developmental patterning in intact roots. The indeterminate growth of the root is driven by the activity of stem cells located at the root apex, housed in a molecular microenvironment where the genetic, hormonal, and metabolic conditions maintain them in an undifferentiated state (Cruz‐Ramírez et al., 2012; Galinha et al., 2007; García‐Gómez et al., 2020, 2021, 2022; Long et al., 2017; Mähönen et al., 2014; Sabatini et al., 1999; Salvi et al., 2020; Strotmann & Stahl, 2021; Tsukagoshi et al., 2010; Weits et al., 2021). The root stem cell niche (SCN) consists of the QC cells, surrounded by different sets of initials cells that produce in a stereotypical radial organization vascular, pericycle, cortex/endodermis, epidermis/lateral root cap, or columella cells (Figure 1a). Stem cells divide asymmetrically to self‐renew and produce progeny that subsequently undergoes multiple rounds of rapid divisions in the meristem, then grow fast and anisotropically in the elongation zone (EZ), and finally, differentiate into the specialized cell types of the mature root.

The above‐mentioned stereotypical spatial organization into different radially positioned cell types as well as distinct longitudinal developmental zones for division, elongation, and differentiation is regulated by numerous, often mobile, molecular regulators that allow cells to coordinate with each other. The radial organization of the root into cell files with distinct cellular fates is, among other factors, regulated by the mobile transcription factor (TF) SHORTROOT (SHR) that is expressed in the stele and then the protein moves outward to the adjacent layer through the plasmodesmata, thereby patterning the endodermis, cortex, and QC cell layers (Cui et al., 2007; Figure 1b). This is mediated by the physical association of SHR with SCARECROW (SCR), JACKDAW, BLUEJAY, and other BIRD TFs (Long et al., 2015) to nuclearly retain SHR in order to prevent further outward movement and enable the regulation of ground tissue‐specific transcriptional programs (Long et al., 2017; Moreno‐Risueno et al., 2015). Particularly, the SHR‐SCR protein complex induces the expression of *SCR* itself, thus setting a positive feedback loop that sequesters SHR in the cell layer surrounding the stele (Cui et al., 2007). Interestingly, SHR orthologues from two monocot species exhibit hypermobility when expressed in the stele of Arabidopsis roots despite strongly binding to Arabidopsis SCR (Wu et al., 2014; Wu & Gallagher, 2014), suggesting the existence of additional layers of regulation in SHR movement. One such layer may involve the regulation of the intracellular trafficking of SHR. The current conceptual model of SHR intracellular transport toward the plasmodesmata proposes that SHR‐associated endosomes travel along the cytoskeleton pausing at the intersection of microtubules and actin filaments in the cell cortex (Spiegelman et al., 2018). Such pausing is mediated by the non‐motile kinesin G (KinG) and has been shown to increase the efficiency of SHR cell‐to‐cell movement (Spiegelman et al., 2018). Thus, KinG, the endomembrane system, and the integrity of the cytoskeleton may constitute an additional layer of regulation in SHR intercellular movement, and thus tissue patterning.

Then, the longitudinal organization of the root apex into developmental zones is regulated by the interplay between the hormones auxin and cytokinin (CK), and the mobile TFs PLETHORA (PLT), where auxin and PLT are distributed in a gradient along the length of the root with a maximum in the SCN, and proximally delimited by an EZ‐centered CK signaling domain (Figure 1; Galinha et al., 2007; Mähönen et al., 2014; Sabatini et al., 1999; Salvi et al., 2020). Due to the acid nature of the auxin molecule and the relatively neutral pH inside the cells, intracellular auxin exists mostly in its deprotonated form limiting its passive cellular auxin export (Paterlini, 2020). This is instead mediated by the PINFORMED (PIN) and ABCB/PGP transporters, as well as direct cell‐to‐cell transport via plasmodesmata (Band, 2021; Bennett et al., 1996; Blilou et al., 2005; Cho & Cho, 2013). In the cell wall, with its lower pH, a significant fraction of auxin is protonated, allowing auxin active import through AUX/LAX membrane transporters and also transmembrane diffusional import (Swarup & Péret, 2012). The PIN auxin exporters, which can have a polar membrane localization, allow for directional auxin transport. Different cell types express different *PINs* with distinct polarity patterns, forming a rootward flux through the stele, connected to a shootward and inward flux in the epidermis/cortex through non‐polar auxin transport in the columella. Together this creates a so‐called reflux loop that generates an auxin gradient with a maximum in the SCN (Grieneisen et al., 2007) that is subsequently fine‐tuned by auxin import (Band et al., 2014), local auxin biosynthesis (Brumos et al., 2018; Santuari et al., 2016) and catabolism (Mellor et al., 2016), CK antagonism (Di Mambro et al., 2017; Salvi et al., 2020), and plasmodesmatal transport (Mellor et al., 2020). The thus generated high auxin levels in the root SCN induce the localized expression of the *PLT* transcription factors (Mähönen et al., 2014). Despite their localized expression, the PLTs form a protein gradient along the meristem due to high protein stability combined with meristematic cell division and plasmodesmata‐mediated cell‐to‐cell diffusion (Mähönen et al., 2014). The slow timescale of the auxin‐induced *PLT* expression ensures the stability of developmental zonation (Mähönen et al., 2014), yet faster regulation is possible through regulating PLT protein stability. For example, the energy signaling regulator TARGET OF RAPAMYCIN (TOR) (Zhang et al., 2022) as well as salt stress (Hao et al., 2023) induce PLT1 and/or PLT2 phosphorylation, resulting in increased protein stability. This results in enhanced PLT protein maxima and gradient length, likely important in the recovery, adaptation, and maintenance of meristem function under diverse environmental conditions.

Specification of the stem cell niche involves the convergence of the radial and longitudinal patterning gradients (Figure 1). SHR‐induced expression of *SCR* in the cell layer that surrounds the stele coincides with a maximum of *PLT* expression in the SCN, enabling the local formation of the PLT1‐SCR‐teosinte‐branched cycloidea PCNA (TCP) protein complex that induces the expression of the QC‐specific and mobile TF WUSCHEL‐related homeobox 5 (WOX5) (Pi et al., 2015; Shimotohno et al., 2018). The indirect PLT‐mediated auxin activation of WOX5 (Shimotohno et al., 2018) combined with its ARF10/16‐mediated repression in the columella initials (Ding & Friml, 2010; García‐Gómez et al., 2017) at least partly explain the localized expression of *WOX5* in the root SCN.

## TYING LOOSE ENDS: TO REGENERATE OR DIFFERENTIATE

**Figure 2:**
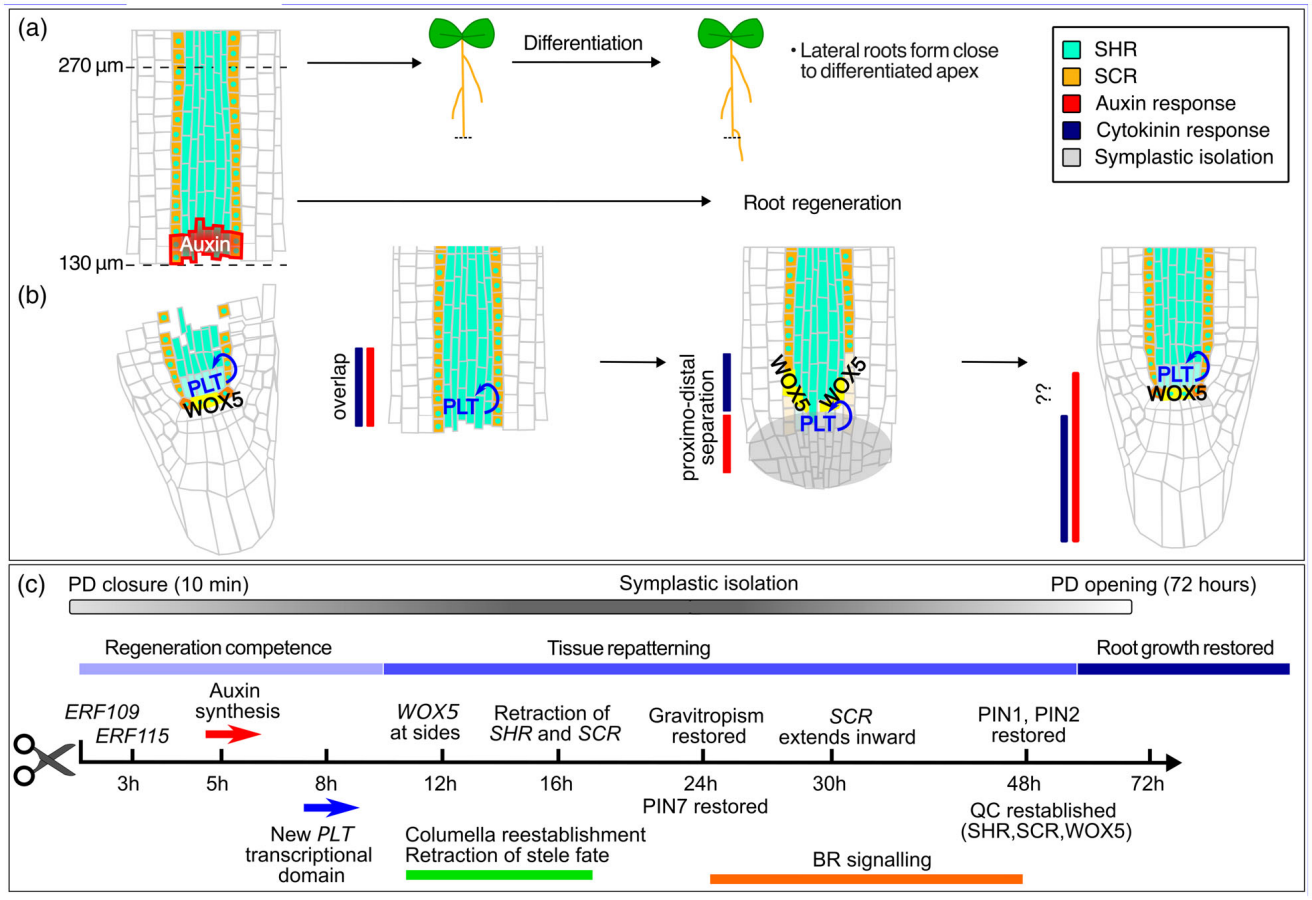
Landmarks of root regeneration. Depending on the height of the cut, either differentiation and formation of lateral roots close to the tip (a) or regeneration will follow (b). In the latter case, first, the root stump gains regeneration competence, and then the tissue gets repatterned until the growth of the root is restored. Tissue repatterning is accompanied by a series of dynamic changes in gene expression and hormonal distribution summarized in (c). BR, brassinosteroids; ERF115, ETHYLENE RESPONSE FACTOR 115; PD, plasmodesmata; PLT, PLETHORA; QC, quiescent center; SCR, SCARECROW; SHR, SHORTROOT; WOX5, WUSCHEL‐related homeobox 5.

While loss of only a few cells is typically successfully resolved through local divisions and fate changes in neighboring cells (Hong et al., 2017; Marhava et al., 2019), in the case of a full root tip resection, the height at which the cut is performed and the extent of damage determine whether or not a new root apex can be regenerated (Durgaprasad et al., 2019; Sena et al., 2009). Indeed, instead of regeneration, full differentiation of the root tip occurs if cuts remove most of the meristematic cells or if the remaining cells are damaged, in which case new lateral roots distal to the cut are formed (Figure 2; Durgaprasad et al., 2019).

So, what determines regeneration success? The removal of the root apex will produce a stump in which molecular factors will remain at diverse levels depending on the location of the cuts and their spatial distribution prior to the cut. For the PLTs, removal of the root apex will result in the loss of their transcriptional domain, yet part of the PLT protein gradient will remain in the stump (Durgaprasad et al., 2019). Low cuts, removing only the columella, SCN, and part of the meristem cells, result in stumps with relatively high remaining PLT levels, allowing a PLT auto‐activation loop to re‐establish the PLT transcriptional domain (Durgaprasad et al., 2019). This response is observed by 8 hours post‐cut (hpc) (Durgaprasad et al., 2019), well before changes in cell fate (>12 hpc, Efroni et al., 2016) or the recovery of PIN polarity patterns (>24 hpc, Sena et al., 2009), and thus can be dubbed as being part of the initial decision in regeneration. High cuts produce root stumps with lower levels of remaining PLT protein, preventing PLT auto‐activation from gaining momentum and hence resulting in low regeneration success (Durgaprasad et al., 2019). Over‐expressing *PLT* for 2 h prior to the cut extends the regeneration competence zone up to the EZ (Durgaprasad et al., 2019). This aligns with previous findings showing that ectopic *PLT* expression in the EZ enables cells to maintain division competence (Mähönen et al., 2014). Surprisingly, over‐expressing *PLT* for longer compromises regeneration of even low cuts, suggesting a bell‐shaped PLT dosage effect on regeneration competence (Durgaprasad et al., 2019). Interestingly, while regeneration requires division competence, the mitotic rate of the cells in the stump drops significantly within the initial 4 h of regeneration (Kral et al., 2016). Moreover, the cell divisions involved in regeneration require a constant influx of sucrose (Omary et al., 2023) and recent research suggests how the energy status of the plant could be integrated into the go–no‐go regeneration decision through TOR‐signaling‐mediated enhanced PLT protein stability (Zhang et al., 2022).

Contrary to the PLTs, the role of auxin in root regeneration competence does not appear to depend on the auxin levels remaining in the stump. Instead, regeneration depends on the capacity to induce local auxin biosynthesis (Matosevich et al., 2020). Initially, auxin levels increase close to the wound in both low and high cuts (Durgaprasad et al., 2019; Matosevich et al., 2020), consistent with computational models predicting auxin accumulation at the cut site due to an interrupted auxin reflux loop (Mironova et al., 2012). Such a rapid auxin accumulation is also observed in local cell damages in the meristem and kickstarts regeneration (Canher et al., 2020; Hoermayer et al., 2020). In root regeneration, a strong and persistent auxin response occurs only in low cuts and involves the expression of auxin biosynthesis enzymes within the first hours after wounding (Matosevich et al., 2020). Indeed, experiments blocking polar auxin transport showed it is dispensable for the initial phases of root regeneration (Matosevich et al., 2020). In contrast, inhibiting overall auxin biosynthesis compromises regeneration competence which can only be restored via localized root auxin supply (Matosevich et al., 2020). Supplying auxin to high‐cut roots extended the regeneration competence zone (Matosevich et al., 2020) yet reached less than half the success rate of low cuts (Matosevich et al., 2020). This suggests that while auxin is necessary, additional longitudinal information remaining in the stump might be needed. This could involve the PLT transcription factors, or other genes whose expression mirrors the progression from stemness to differentiation along the meristem (Wendrich et al., 2017).

Regeneration should occur exclusively in the presence of tissue damage. The ETHYLENE RESPONSE FACTOR 115 (ERF115) is a key wound response factor initially identified as a rate‐limiting factor for QC divisions (Heyman et al., 2013), and positively regulated by reactive oxygen species (ROS) in the context of intact roots (Kong et al., 2018). Wound‐induced pressure changes rapidly activate *ERF115* expression in cells next to damaged cells (Canher et al., 2020; Heyman et al., 2016; Hoermayer et al., 2020; Matosevich et al., 2020). ERF115 promotes auxin responses through inducing auxin biosynthesis (Matosevich et al., 2020) and the expression of the auxin response factor *ARF5/MONOPTEROS* (Canher et al., 2020), while its own maintained expression is auxin dependent (Canher et al., 2020). The ERF115‐dependent local increase in auxin responses may explain why polar auxin transport is initially dispensable for root tip regeneration, whereas the successful induction of auxin biosynthesis only occurring in low cuts again suggests an integration with remaining longitudinal patterning information (Matosevich et al., 2020). Additionally, ERF115 promotes divisions by binding the SCR inhibitor RETINOBLASTOMA (RBR) (Zhou et al., 2019), while its association with PHYTOCHROME A SIGNAL TRANSDUCTION1 (PAT1) and other GRAS TFs drives the expression of the cellular reprogramming regulator WOUND‐INDUCED DEDIFFERENTIATION1 (WIND1) (Bisht et al., 2023; Heyman et al., 2016).

In addition to pressure changes, jasmonic acid (JA), a defense hormone involved in responses to necrotrophic pathogens, herbivores, as well as other environmental insults that affect the integrity of the plant, is involved in ERF115 regulation (Ghorbel et al., 2021; Hewedy et al., 2023; Zhou et al., 2019). JA accumulates within minutes after damage and rapidly upregulates ERF109, which in turn activates ERF115 and CYCD6;1 (Zhou et al., 2019). JA‐induced *ERF109* expression is much stronger in low cuts, again indicating integration with longitudinal patterning information (Zhou et al., 2019). While *ERF109* displays a broad expression in the root, JA‐induced *ERF115* expression occurs mainly in the QC and protoxylem (Zhou et al., 2019), consistent with cut‐induced auxin responses initially being observed in the protoxylem (Matosevich et al., 2020). Interestingly, both JA and ROS promote *ERF115* expression in intact roots (Kong et al., 2018; Zhou et al., 2019), and also antagonize auxin by repressing *PLT1* and *PLT2* expression (Chen et al., 2011; Kong et al., 2018). We hypothesize that under limited damage, JA‐ and ROS‐induced ERF115 pitches in on the positive feedback between auxin and PLTs, by further enhancing auxin production and signaling, while at higher levels of damage, the JA/ROS‐mediated repression of PLTs combined with their other growth‐ and development‐repressive effects (Guo et al., 2018; Kong et al., 2018; Major et al., 2017; Suzuki & Mittler, 2012; Tsukagoshi et al., 2010; Vega‐Muñoz et al., 2020; Zhang et al., 2023) block the regeneration response. This would enable the plant to integrate the level of damage into the regeneration/defense differentiation decision. Interestingly, a 30‐min exposure to a weak electric field can increase the regeneration competence of high‐cut root stumps (Kral et al., 2016). The enhanced regeneration competence involves a transient reduction of mitotic index and an increase in auxin response levels (Kral et al., 2016). The enhanced regeneration may possibly occur through the electric field mimicking wound‐induced GLR‐mediated electrical signals mildly stimulating JA and/or ROS signaling, thus promoting regeneration over defense responses (Mousavi et al., 2013; Vega‐Muñoz et al., 2020).

Tissue damage increases local vulnerability to pathogens, and rapid defense and/or differentiation is likely to provide a faster means to seal off tissue from intruders than regeneration. It is thus not surprising that salicylic acid (SA), another major plant defense hormone that stimulates the immune responses to biotrophic pathogens (van Butselaar & van den Ackerveken, 2020), impacts regeneration potential. SA is involved in complex crosstalk with JA (Caarls et al., 2015, 2017; Pieterse et al., 2012; van Wees et al., 2000). In leaves, wounding leads to the rapid release of amino acids that activate glutamate receptors (GLRs) (Bellandi et al., 2022), with the resulting downstream Ca^2+^ fluxes being integrated with the JA signaling discussed above to induce either defense or repair (Vega‐Muñoz et al., 2020). While this glutamate release upon wounding remains to be described in root regeneration, it has been shown that GLR signaling promotes defense over regeneration in roots by inducing SA‐responsive genes that affect wounding‐induced Ca^2+^ fluxes and downstream callose deposition, resulting in reduced cell division rates (Hernández‐Coronado et al., 2022). This SA pathway thus further enhances the bias toward defense and differentiation under broad tissue damage. SA‐mediated enhanced plasmodesmatal closure may have a dual effect. In callus regeneration, symplastic isolation of cells was found to correlate with acquisition of regenerative potential (Godel‐Jedrychowska et al., 2020). However, SA‐treated roots have an altered radial patterning likely due to impaired SHR movement (Pasternak et al., 2019), implying that only a limited or transient plasmodesmatal closure would be beneficial. Interestingly, SA response increases the farther the cells are from the root tip (Hernández‐Coronado et al., 2022), which may explain the decrease in regeneration success with cell age in Arabidopsis.

Ultimately roots must “decide” whether to attempt regeneration based on the current state of the cells, the extent of the damage, and the external conditions. In this decision, ERF115 represents a rapid local signal for the presence of damage, the PLTs provide information about the context and level of stemness of the cells remaining in the stump, while SA and JA levels encode information about the extent of damage and the ongoing biotic and abiotic risks the plant is exposed to. Indeed, while low cuts have the highest regeneration potential, they do not regenerate if there is extended damage and cell death in the stump (Durgaprasad et al., 2019). Similarly, penetration by parasitic nematodes causes extensive meristem damage triggering *ERF115* expression necessary for the development of galls needed for nematode reproduction, yet does not induce regeneration (Ribeiro et al., 2023).

## A CHOREOGRAPHY OF CELL FATE CHANGES REPATTERNS THE ROOT STUMP

Once the immediate wound responses have been triggered, and a new high PLT–high auxin domain providing regeneration competence has been established (from 8 hpc onward), the repatterning process of the stump begins (>12 hpc). Recreating the lost SCN involves a dynamic reprogramming of the fate of the cells remaining in the stump to recreate the stereotypical spatial pattern of the root SCN. Clonal analysis has revealed that stele cells are the main source for the regeneration of the root SCN and columella, while endodermal cells mostly fuel the regeneration of the missing epidermis and lateral root cap (Efroni et al., 2016). Interestingly, the contribution of cell types to regeneration changes with the relative position of the cells (Efroni et al., 2016) and depending on the activity of the polar auxin transport (Matosevich et al., 2020). Namely, the pericycle cells that contribute to endodermis/cortex regeneration in low cuts also form epidermis/lateral root cells in high cuts (Efroni et al., 2016). On the other hand, while endodermal cells mostly regenerate the epidermis and lateral root cap cells, they have been reported to regenerate the SCN and the columella in NPA‐treated low‐cut roots (Matosevich et al., 2020). Single‐cell transcriptomics revealed that within 3 h after a low cut, around 10% of the analyzed stele cells display expression patterns resembling those of columella tissue (Efroni et al., 2016), suggesting their rapid respecification into columella cells and latter reprogramming of other tissues. Yet a functional columella is re‐established much later as root gravitropic responses take much longer to be recovered (55% and 89% of root stumps bend downward in 48 or 72 hpc, respectively) (Sena et al., 2009). It is tempting to think that the fast transcriptional changes conveying a columella fate might be an immediate response of the cells in the outermost layer to the release of cellular pressure and their exposure to the surface, as has been described for regenerative responses in leaves (Iida et al., 2023).

Then, at 16 hpc, a larger proportion of originally stele cells reprogram their gene expression to form further columella, or become either epidermis or ground tissue (Efroni et al., 2016). At this time point the expression of the *SHR* and *SCR* root tissue markers has shifted proximally (Figure 2), accompanied by the faint expression of *WOX5* in the most rootward *SCR*‐expressing cells at 6 hpc (Efroni et al., 2016). We propose that this modified WOX5 domain arises from localized PLT elevation levels (Durgaprasad et al., 2019) combined with SCR persistence. The retracted *SHR* and *SCR* expression domains probably serve to accommodate the new columella to properly repattern the inner and outer root tissue domains as evidenced by the non‐complementary expression patterns of WOL (stele) and WIP4 and PET111 (columella) in the distal cells of the stump (Efroni et al., 2016). After their proximal shift, the radial patterning genes SHR and SCR slowly recreate the stereotypical organization of the root SCN into its characteristic U‐shaped domain of *SCR* expression and SHR nuclearization by 48 hpc (Efroni et al., 2016). As the *SCR* expression extends downward and to the center of the root, *WOX5* expression retracts from the endodermis and becomes confined to the SCN, essentially following the most rootward domain of *SCR* expression (Efroni et al., 2016). We hypothesize that through inducing WOX5‐PLT complex formation, WOX5 might enable the expression of auxin biosynthesis enzymes as observed in callus regeneration (Zhai & Xu, 2021), as well as regulating the fate of the distal columella cells as observed in intact roots (Burkart et al., 2022).

The gradual regeneration of the root SCN involves the gradual confinement of *WOX5* expression to the new QC cells, a process that is affected by cut height and polar auxin transport. The higher the cut is performed, the further apart SCR‐positive endodermal cells are and hence the longer it takes for the U‐shape to be reformed and WOX5 to be confined in the new QC (Sena et al., 2009). Furthermore, WOX5 fails to become confined to the center of the regenerating stump and instead remains expressed for up to 4 days at the sides of the stele in root stumps treated with the PIN inhibitor NPA (Sena et al., 2009), reminiscent of the pattern in NPA‐treated intact roots (Sabatini et al., 1999). Indeed, NPA‐treated stumps display a correct SCR pattern in a U‐shaped domain, albeit with a slight delay (Matosevich et al., 2020). This strongly suggests that while polar auxin transport is dispensable for early phases of root tip regeneration and the patterning of the cell layer surrounding the stele (Matosevich et al., 2020), it is necessary for the correct repatterning of the SCN (Sena et al., 2009). Supporting this idea, the default *PIN7* expression in the stele and the columella but not the SCN is re‐established by 24 hpc, resulting in a more central localized auxin maximum, while normal PIN1 and PIN2 patterns have formed by 48 hpc (Sena et al., 2009). Thus, to refocus WOX5 and form a new QC, PLT and SCR have to be aided by correct auxin transport patterning. Parallel to the role of the PIN efflux transporters, the auxin influx transporters AUX1/LAX are indispensable for regeneration from 24 hpc onward (Matosevich et al., 2020).

In addition to its dependence on auxin, the confinement of *WOX5* expression to the new SCN can be delayed or compromised by changes in the availability and distribution of other hormones including brassinosteroids (BR) and CK. BR signaling is highly induced at 24 hpc and while dispensable for regeneration, it affects the efficiency of the repatterning process (Takahashi & Umeda, 2022). BR biosynthesis inhibition results in a broadened *WOX5* expression domain and a delayed confinement to the new QC (Takahashi & Umeda, 2022). In intact roots, BR regulates the expression of *PIN3*, *PIN4*, and *PIN7* in the columella (Lee et al., 2015). Given the correlated timing of maximum BR signaling with changes in PIN7 expression and polarity (Sena et al., 2009; Takahashi & Umeda, 2022), it appears likely that BR signaling is involved in re‐establishing the auxin reflux loop thereby helping to reposition WOX5. Conversely, CK overlaps with auxin signaling early on in regeneration, and from 12 hpc onward, their responses separate such that auxin is found in the most distal cells and CK in the proximal ones (Figure 2b; Efroni et al., 2016). Interestingly, CK and auxin responses overlap in the columella in intact roots (Liao et al., 2015; Zürcher et al., 2013), and then it is expected that at advanced stages of regeneration, their responses will overlap there again. The spatio‐temporal distribution of auxin and CK in root regeneration (Figure 2b) recapitulates their dynamics in the hypophysis, the extra‐embryonic cell whose asymmetric division produces the QC and the columella during embryogenesis (Müller & Sheen, 2008). Exogenous auxin and/or CK treatments have shown that the spatial separation of their responses is important for the correct patterning of the tissue within the first 24 hpc (Efroni et al., 2016). The relatively small proportion of stele cells with mixed QC–columella cell fate characteristics observed during the initial stages of regeneration could be related to this recapitulation of embryonic development (Efroni et al., 2016). In addition, it may also involve the time required to recover the expression and nuclearization of SHR in the new QC, as this mobile regulator is key in controlling the cell fate transition from QC to columella initials in intact roots (García‐Gómez et al., 2020).

Indeed, both in the development of regeneration competence and in subsequent repatterning, movement through the plasmodesmata of mobile factors like PLT, SHR, WOX5, and auxin plays key roles (Mähönen et al., 2014; Mellor et al., 2020; Pi et al., 2015). Wound signaling influences plasmodesmatal aperture and this has been shown to be important for cell reprogramming in regeneration (Cohen et al., 2023; Godel‐Jedrychowska et al., 2020). Specifically, during regeneration, several lateral organ boundary (LBD) genes are transiently induced, thereby gradually promoting the symplastic isolation of the cells close to the wound (Cohen et al., 2023). Failure in the formation of this isolated cell domain in an *lbd* multiple mutant compromises many key aspects of repatterning. The confinement of auxin responses to the distal cells, the proximal retraction of *SHR* expression, the re‐establishment of expression of *WOX5* in the central cells, the specification of the outer columella cells, and the re‐establishment of gravitropic responses all appear affected (Cohen et al., 2023). Which of these effects are a direct consequence of persistent cell–cell movement of molecular regulators and which are more indirect effects of other processes being affected is not yet clear. An interesting starting point for future research is the fact that the formation of a symplastically isolated domain prevents entry of the mobile transcription factor SHR (and many other mobile regulators) which instead will accumulate in the cells at the boundary of the domain.

## TOWARD A MULTI‐SCALE UNDERSTANDING OF ROOT REGENERATION

**Figure 3:**
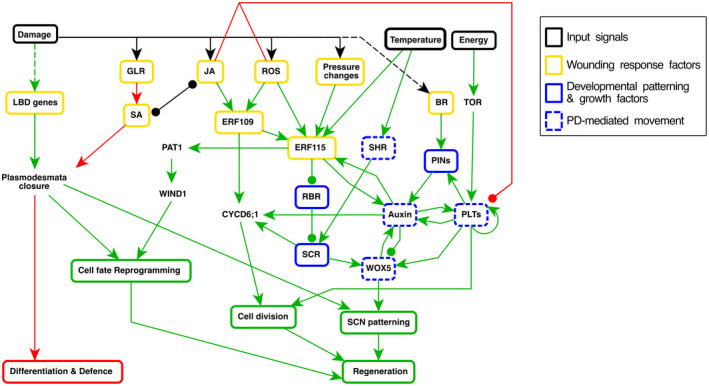
Regulatory network underlying root tip regeneration. The regulators and their interactions mentioned in this review are part of a complex regulatory network defining if regeneration (green) or differentiation (red) will take place. In addition to the shown interactions, auxin also affects plasmodesmata (PD) closure (Han et al., 2014). For simplicity, here we only include the interactions discussed in this review. Notice that the damage response regulators JA and ROS are both inducers of ERF109 and ERF115 (green arrows), and repressors of PLT1,2 (red arrows). BR, brassinosteroids; ERF115, ETHYLENE RESPONSE FACTOR 115; GLR, glutamate receptor; JA, jasmonic acid; LBD, lateral organ boundary; PIN, PINFORMED; PLT, PLETHORA; RBR, RETINOBLASTOMA‐RELATED; ROS, reactive oxygen species; SA, salicylic acid; SCN, stem cell niche; SCR, SCARECROW; SHR, SHORTROOT; WOX5, WUSCHEL‐related homeobox 5.

Once the competence to regenerate decision has been taken, the cells remaining in the stump have obtained high levels of PLTs and auxin and are competent to divide. The subsequent occurrence of the fate reprogramming and growth repatterning process suggests that elevated auxin and PLT levels and division capacity may be driving this repatterning. Supporting this idea, clear links exist among auxin, PLTs, and *WOX5* expression (Figure 3, section Setting the Stage). Additionally, links have been suggested between the PLTs and division orientation (Du & Scheres, 2017; Rodriguez et al., 2015). In contrast, how the repatterning of the PIN reflux network, SHR, SCR, and columella cell fate factors is related to the elevated auxin and PLTs, or how this together with divisions leads to a restoration of the typical U‐shaped endodermis and SCR pattern, is far less clear. Additionally, exactly how the remaining PLT levels and other remaining molecular factors integrate with ERF115 wound signaling, SA signaling, and auxin response to modulate regeneration success depending on the height of the cut, or why the contribution of cell types to regeneration depends on cut height and the polar auxin transport (Efroni et al., 2016; Matosevich et al., 2020), remain open questions.

Importantly, SCR, through forming a complex with PLT and TCP, impacts PLT‐mediated WOX5 patterning (Shimotohno et al., 2018). Similarly, PIN type and polarity localization affect auxin and PLT patterns, while cell fate likely affects PIN type and polarity pattern (Xu et al., 2006). Also, among auxin, ERF115, JA, SA, and PLTs, a complex network of mutual regulations exists (Figure 3). Additionally, PLTs, SHR, and WOX5 are all mobile transcription factors traveling through plasmodesmata, whereas their co‐occurrence in cells leads to protein complex formation, DNA binding and gene regulation, and restriction of further cell‐to‐cell movement (Burkart et al., 2022; Cui et al., 2007; Shimotohno et al., 2018; Zhai & Xu, 2021). Wound signaling impacts plasmodesmatal closure, thereby affecting cell–cell signaling. In addition, SHR intercellular movement relies on the cytoskeleton (Spiegelman et al., 2018; Wu & Gallagher, 2013), and then microtubule rearrangements caused by wounding (Hamant et al., 2008; Hoermayer et al., 2024) not only affect division orientation but may also transiently impact how cells exchange molecular information and possibly how the tissue gets repatterned. Thus, molecular regulatory networks in the cells, intracellular trafficking dynamics, intercellular communication, and tissue‐level growth and division dynamics are processes occurring at different spatial scales that impact the different phases of the regeneration process (Figure 2). It is this kind of complex, often bi‐directional regulatory interactions and intracellular, intercellular, and tissue‐level processes that necessitate the use of multi‐scale models to unravel the mechanisms underlying root tip regeneration.

Previous research in intact roots has demonstrated the power of combining computational modeling with experimental research, revealing how the combined activity of a suite of molecular regulators drives root SCN patterning, auxin gradient formation, developmental zonation, postembryonic development, and lateral root pre‐patterning (e.g., García‐Gómez et al., 2020, 2022; Grieneisen et al., 2007; Mähönen et al., 2014; Mironova et al., 2012; Salvi et al., 2020; van den Berg et al., 2021). In the context of root regeneration, this approach can be harnessed to integrate the different pieces of the root regeneration puzzle, summarized here, into multi‐scale computational models. Through integrating known players and their interactions, cell‐to‐cell communication, and cell division and growth, these models enable us to assess the emergent consequences of incorporated processes, test novel hypotheses *in silico*, and generate predictions that can be tested experimentally. Creation of these models would require the integration and/or combined application of several existing modeling approaches as well as certain extensions. The first logical step would be to combine root tip models for intracellular and tissue‐level hormonal patterning and limited gene expression (e.g., Di Mambro et al., 2017; Moore et al., 2024; Salvi et al., 2020), with models with a detailed description of gene expression and patterning (Cruz‐Ramírez et al., 2012; García‐Gómez et al., 2017, 2020). Next, these should be combined with models describing the mechanics of root growth dynamics (Marconi et al., 2021). However, here significant extensions in both models and knowledge are needed to explain the more complex and variable division orientations in the regenerating tissue and surrounding the QC. When it comes to modeling cytoskeleton‐mediated dynamics or trans plasmodesmata transport, models exist (Chakrabortty et al., 2018; Deinum et al., 2019; Jacobs et al., 2022) that can be extended to also consider the trafficking of SHR endosomes, but their level of detail and computational demands prohibit one‐on‐one integration in tissue‐level models. Instead, simpler “rules” should be derived from these models that enable implementation in tissue‐level multi‐scale models. Finally, recent insights on the importance of energy level signaling in developmental growth and patterning (Stitz et al., 2023; Zhang et al., 2022) suggest that we may need to develop both standard root models as well as models for root tip regeneration that include the influence of physiology on development.

A further challenge that modeling needs to address is why tissue patterns in regenerating roots are far more variable than those of intact mature roots. Apart from starting the simulations from slightly different anatomical tissue layouts when applying *in silico* cuts, this may require the incorporation of stochasticity in gene expression, cell–cell signaling, and cell growth and division dynamics in our simulations. Importantly, models are of immense use even if important aspects of our knowledge are still missing. As an example, even if we do not yet understand how retraction of certain cell fate factors is driven, simply “painting” the observed gene expression patterns at a regenerating root “canvas” at a certain time point of regeneration, we can study subsequent steps and downstream emergent processes. This enables us to investigate different stages of regeneration independently, before tying them together into an overall mechanistic model completely capable of describing all steps from incorporated regulatory mechanisms. Ultimately, they allow us to demonstrate whether the incorporated processes are necessary and sufficient or, more likely, that we are still missing essential pieces of the puzzle.

Some hints on missing puzzle pieces may come from existing knowledge. As an example, earlier work suggests that an additional source of positional information might be provided by mature meristematic cells toward the initial cells to perpetuate the tissue pattern in the root apex (van den Berg et al., 1995). Thus, a combination of persistent and/or transient positional signals could help by forming a sort of coordinate system guiding cell fate changes to reconstitute the missing tissues in root regeneration. Although the nature of these signals is still undefined, they could be a combination of the genes that are expressed in a gradient pattern along the meristem (Wendrich et al., 2017). As another example, suboptimal high temperatures increase *ERF115* expression in intact roots (Heyman et al., 2013), and grafting is known to be aided by temperature (Feng et al., 2023). Modeling would be useful to integrate the role of environmental signals in regeneration, for example, by determining whether known effects of the root temperature–response pathways on auxin (Ai et al., 2023) and *SHR* expression (Perez‐Garcia et al., 2023) could explain these observations. Still, iterating between modeling and experiments will be essential to identify undoubtedly more missing pieces and thus solve the regeneration puzzle.

We end by pointing out some remaining challenges in the two individual fields that need to be met to maximize the power of a combined experimental‐modeling approach. For experiments, challenges lie in the number of genes and hormone activity that ideally should be tracked simultaneously, as well as in tracing over time the fates of individual cells. For the former, spatial transcriptomics and the development of combinatorial sensors integrated with sophisticated mapping appear promising tools (Birnbaum et al., 2022), while the latter requires the live imaging of regenerating roots on a sufficiently high temporal resolution to enable cell tracking. For modeling, challenges lie in truly integrating models for hormonal–genetic patterning (García‐Gómez et al., 2017, 2020; Mironova et al., 2012; Salvi et al., 2020; van den Berg et al., 2021) with those of mechanics of growth (Bassel et al., 2014; de Vos et al., 2014; Kelly‐Bellow et al., 2023; Weise & ten Tusscher, 2019), an integration that has thus far only been achieved to a limited extent (Marconi et al., 2021), the integration of physiological processes such as energy status within developmental decision‐making processes (Liu et al., 2020), and ultimately the parametrization of these increasingly complex models. On a more general level, a challenge lies in obtaining from high‐dimensional data provided by modern omics experiments a core network of key players that enable explaining the phenomenon of interest while keeping models tractable and understandable.

**OPEN QUESTIONS**

1. How does the molecular information remaining in the stump inform the spatio‐temporal order of cell fate changes in regeneration?
2. How does the energy status of the plant influence the decision for root tip regeneration versus differentiation and distal lateral root formation?
3. What is the relative importance of cell lineage and position for the contribution a cell type will have in regeneration?
4. What role do cell fate reprogramming of existing cells and formative asymmetric divisions play in the repatterning of the root stump?
5. How do abiotic conditions (i.e., temperature and nutrient presence) alter the success of root regeneration?

## CONCLUSION

In plants, regeneration occurs in response to both limited, localized damages of one or a few cells and resection of entire root tips, which can occur as a result of, for example, cold stress, nematode penetration, soil compaction, and herbivory. Here, we focused on the more extreme case of root regeneration after root tip resection, summarizing and connecting existing knowledge, and pinpointing major open questions. The removal of the root apex causes a dramatic alteration of the tissue microenvironment due to the interruption of auxin fluxes in the tissue (Mironova et al., 2012), the activation of wound signaling in the vicinity (Zhou et al., 2019), a distal shift in the relative position of expression of root patterning regulators (Efroni et al., 2016), and subsequent repatterning. Some of these changes in positional information persist for hours (e.g., *SHR* and *SCR* expression patterns), others are transient due to the rapid redistribution of molecular information (e.g., auxin), and yet others will gradually build up (e.g., PLT levels through its auto‐activation). We suggest integration of experiments with multi‐scale modeling as a much‐needed step forward to unravel how these many multi‐level, intertwined processes involved together enable self‐organized root tip regeneration.

## AUTHOR CONTRIBUTIONS

MLG‐G and KTT wrote the manuscript. Both authors contributed to the article and approved the submitted version.

## CONFLICT OF INTEREST STATEMENT

The authors declare that they have no conflicts of interest associated with this work.
